# Quantitative lateral flow strip assays as User-Friendly Tools To Detect Biomarker Profiles For Leprosy

**DOI:** 10.1038/srep34260

**Published:** 2016-09-29

**Authors:** Anouk van Hooij, Elisa M. Tjon Kon Fat, Renate Richardus, Susan J. F. van den Eeden, Louis Wilson, Claudia J. de Dood, Roel Faber, Korshed Alam, Jan Hendrik Richardus, Paul L. A. M. Corstjens, Annemieke Geluk

**Affiliations:** 1 Dept. of Infectious Diseases, Leiden University Medical Center, The Netherlands; 2 Dept. Molecular Cell Biology, Leiden University Medical Center, The Netherlands; 3 Department of Public Health, Erasmus MC, University Medical Center Rotterdam, Rotterdam, The Netherlands; 4 Rural Health Program, The Leprosy Mission International Bangladesh, Nilphamari, Bangladesh

## Abstract

Leprosy is a debilitating, infectious disease caused by *Mycobacterium leprae.* Despite the availability of multidrug therapy, transmission is unremitting. Thus, early identification of *M. leprae* infection is essential to reduce transmission. The immune response to *M. leprae* is determined by host genetics, resulting in paucibacillary (PB) and multibacillary (MB) leprosy associated with dominant cellular or humoral immunity, respectively. This spectral pathology of leprosy compels detection of immunity to *M. leprae* to be based on multiple, diverse biomarkers. In this study we have applied quantitative user friendly lateral flow assays (LFAs) for four immune markers (anti-PGL-I antibodies, IL-10, CCL4 and IP-10) for whole blood samples from a longitudinal BCG vaccination field-trial in Bangladesh. Different biomarker profiles, in contrast to single markers, distinguished *M. leprae* infected from non-infected test groups, patients from household contacts (HHC) and endemic controls (EC), or MB from PB patients. The test protocol presented in this study merging detection of innate, adaptive cellular as well as humoral immunity, thus provides a convenient tool to measure specific biomarker profiles for *M. leprae* infection and leprosy utilizing a field-friendly technology.

Leprosy, a chronic infectious disease caused by *Mycobacterium leprae* (*M. leprae*) ranking second as the most pathogenic mycobacterial infectious disease after tuberculosis (TB), is still considered a major threat in developing countries^1^. The condition is characterized by skin lesions and damage to peripheral nerves, the hallmark of leprosy pathology often resulting in severe, life-long disabilities and associated stigma^2^,^3^.

Despite the remarkable decrease in prevalence following introduction of multidrug therapy, it remains challenging to further reduce transmission as substantiated by the stable global annual incidence around 200,000 new cases for the past 10 years^4^. This continued transmission is largely due to *M. leprae* infected individuals lacking clinical symptoms^5^. In addition, identification of host-derived biomarkers for progression to disease is complicated by the low incidence and long incubation time requiring extensive, longitudinal studies. Furthermore, although molecular techniques to elicit strain differences within the leprosy bacillus are important diagnostic tools to enhance our understanding of the epidemiology of leprosy, differentiate between relapse and re-infection^6^,^7^,^8^,^9^,^10^, these pathogen-derived profiles are not suitable to indicate development of leprosy in infected, asymptomatic individuals.

These hurdles contributed to the current lack of tests for detection of asymptomatic *M. leprae* infection and diagnosis of early stage leprosy^11^. As clinical resistance to commonly used antibiotics in leprosy treatment is increasingly occurring^12^,^13^, such tests should be highly specific to prevent redundant use of antibiotics.

Clinical manifestations closely parallel cellular immunity to *M. leprae* such that leprosy presents as a characteristic spectrum ranging from tuberculoid (TT) or paucibacillary (PB) leprosy to lepromatous (LL) or multibacillary (MB) leprosy^14^. TT patients in general show strong T helper 1 cell (Th1) immunity with exacerbated levels of pro-inflammatory cytokines and develop localized granulomatous disease with bacilli scarcely detectable in their lesions. At the opposite pole of the spectrum are LL patients who predominantly generate Th2 and anti-inflammatory cytokines such as interleukin-10 (IL-10) resulting in disseminating, progressive infections^15^. In between these two opposite poles of the leprosy spectrum, borderline states of leprosy [borderline tuberculoid (BT), borderline (BB) and borderline lepromatous (BL)] are positioned. Due to the diverse disease spectrum, detection of *M. leprae* infection in diagnostic tests requires multiple, diverse biomarkers specific for both cellular and humoral mediated immunity. In previous studies we have shown that IFN-γ-inducible protein 10 (IP-10) in response to a *M. leprae*-specific antigen (ML2478) correlates with *M. leprae* exposure and thereby the risk of infection and its subsequent transmission^16^. Additionally, we demonstrated that chemokine (C-C motif) ligand 4 (CCL4), a component of the innate immunity, can be used to identify pathogenic immunity against *M. leprae* since it was increased in patients, partly in household contacts but not in endemic controls^16^. IL-10, on the other hand, is associated with suppression of Th1 cells in leprosy^17^,^18^,^19^. Moreover, most lepromatous patients with high bacillary loads produce antibodies against the *M. leprae* specific phenolic glycolipid I (PGL-I)^20^,^21^, which are hardly detected in PB^22^. Hence, sensitive tests that can simultaneously quantitate multiple analytes in one sample provide apt tools to characterize different clinical leprosy types. In particular, tests based on multicomponent host biomarker profiles that can identify *M. leprae* infected individuals (yet) without clinical symptoms of leprosy, will be useful for guidance of prophylactic treatment, thereby contributing to reduction of *M. leprae* transmission as well as prevention of disabilities.

Inherent to the situation in leprosy endemic areas is the absence of sophisticated laboratories. It is therefore imperative that new diagnostic tests are facilitated for application in the field. Up-converting phoshor lateral flow assays (UCP-LFAs) have previously shown to be robust, low-complexity assays, representing a field-friendly alternative for common laboratory-based ELISAs^23^,^24^, applicable for detection of multiple pathogens including food-borne pathogenic strains and potential biowarfare/bioterrorism agents^25^,^26^,^27^. Field evaluation of UCP-LFAs for detection of IL-10, IP-10, CCL4 and anti-PGL-I IgM demonstrated high correlation with ELISAs using samples from cohorts of limited numbers of leprosy- or TB patients^28^,^29^.

In the current study UCP-LFAs were applied to a more extensive (five-fold) sample size compared to our previous studies, derived from a randomized BCG vaccination field trial in Bangladesh^30^. Six test groups were included: MB patients, PB patients, healthy household contacts (HHC), HHC vaccinated with Bacillus Calmette-Guérin (BCG) (HHC&BCG), HHC who developed leprosy after BCG vaccination (new cases; NC) and endemic controls (EC) from the same area without known contact with leprosy patients. This extended cohort study allowed exploratory identification of biomarker profiles for *M. leprae* infection, leprosy disease per se, the type of leprosy and BCG vaccination as determined with UCP-LFAs for the above indicated targets.

## Results

### Performance of the UCP-LFA versus ELISA

Whole blood samples (n = 726) from all individuals were analysed using ELISA, as well as the field-friendly UCP-LFAs for IL-10, IP-10, CCL4 and anti-PGL-I antibodies. Comparison of UCP-LFA and ELISA results demonstrated significant correlation for all four biomarkers (p < 0.0001), confirming earlier observations^28^,^29^.

**Figure 1:**
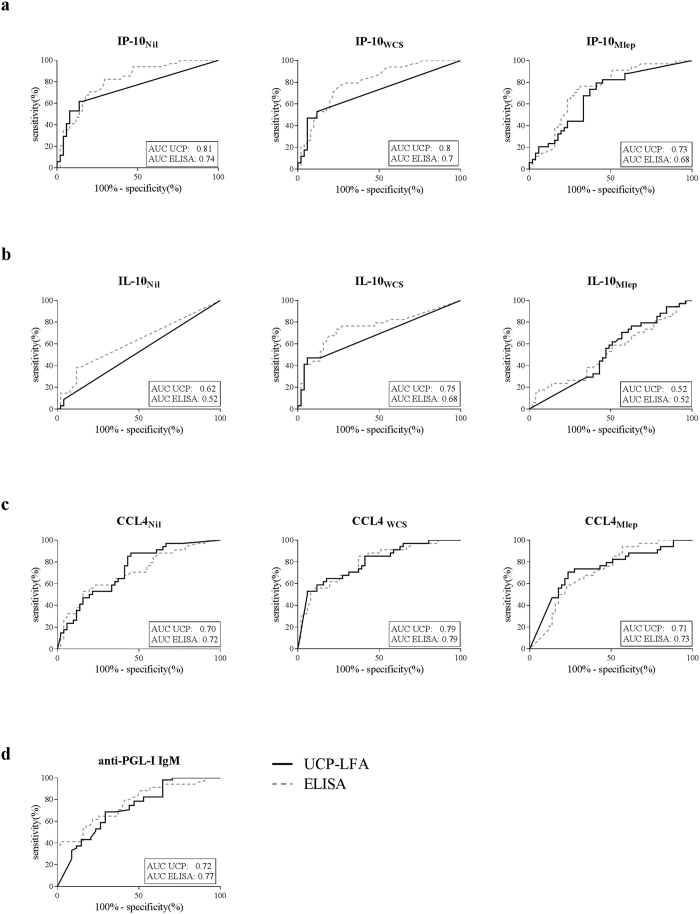
Discriminatory capacity of ELISA and UCP-LFA. To compare the ability of ELISA (dotted line) and UCP-LFA (solid line) to discriminate between individuals with or without disease ROC curves were computed using data of MB patients and EC. Areas under the curve (AUCs) were compared for all 10 conditions tested, shown in the lower right corner of each graph. (**a**) ROC curves for IP-10 stimulated and unstimulated samples based on concentration in pg/ml, showing an improved AUC for the UCP-LFA for IP-10_Nil_ and IP-10_WCS_. (**b**) ROC curves for IL-10 stimulated and unstimulated samples based on concentration in pg/ml, showing an improved (IL-10_Nil_) or equal AUC for UCP-LFA. (**c**) ROC curves for CCL4 stimulated and unstimulated samples based on concentration in pg/ml, showing comparable values for UCP-LFA and ELISA. (**d**) ROC curves for anti-PGL-I IgM in unstimulated samples based on ratio, showing comparable AUCs for ELISA and UCP-LFA.

The diagnostic performance of the UCP-LFA in comparison to ELISA was further assessed through AUCs for the two most distinct phenotypes: MB patients (n = 34) and EC (n = 51). IL-10, IP-10 and CCL4 levels were determined in Nil, WCS and Mlep samples, as well as anti-PGL-I IgM levels (Fig. 1). The IL-10 and IP-10 UCP-LFAs outperformed the corresponding ELISAs, whereas the CCL4 and anti-PGL-I IgM tests performed equally. For discrimination of MB patients from EC, the proposed diagnostic field-tool UCP-LFA provides an equally well or even better alternative for the conventional ELISAs.

### *M. leprae* specific responses based on single analyte UCP-LFA measurements

**Figure 2:**
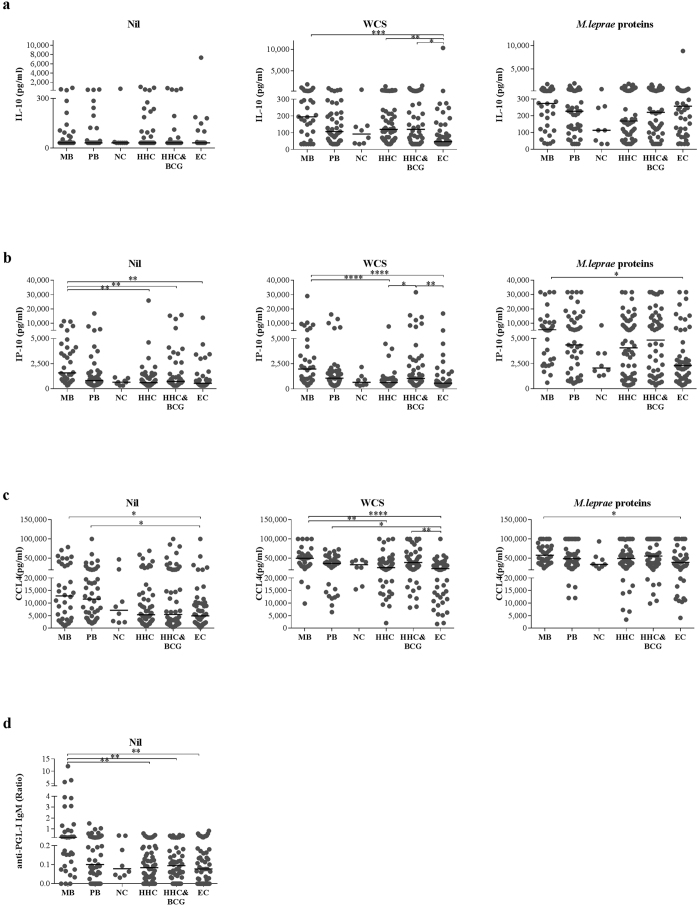
Identification of *M. leprae* specific IL-10, IP-10, CCL4 and anti-PGL-I IgM antibodies by UCP-LFA. (**a**) IL-10 concentrations (pg/ml) measured per group per stimulus show that MB patients, HHC and BCG-vaccinated HHC significantly differ from EC upon WCS stimulation. (**b**) IP-10 concentrations (pg/ml) measured per group per stimulus show that MB patients significantly differ from EC in both stimulated and unstimulated samples, from HHC in unstimulated and WCS stimulated samples and from BCG vaccinated HHC in unstimulated samples. BCG vaccinated HHC significantly differ from HHC and EC upon WCS stimulation. (**c**) CCL4 concentrations (pg/ml) measured per group, per stimulus show that MB patients significantly differ from EC in both stimulated and unstimulated samples and from HHC in WCS stimulated samples. PB patients significantly differ from EC in unstimulated and WCS stimulated samples and BCG vaccinated HHC significantly differ from EC in WCS stimulated samples. (**d**) anti-PGL-I IgM ratio measured per groups shows that MB patients have significantly higher levels of anti-PGL-I IgM compared to HHC, BCG vaccinated HHC and EC. P-values: *p ≤ 0.05, **p ≤ 0.01, ***p ≤ 0.001, ****p ≤ 0.0001.

In order to put the quantitative test results obtained with the four single UCP-LFAs in the context of their biomarker potential, we assessed each analyte/stimulus combination by comparing median group levels. As indicated by the AUCs in Fig. 1, MB patients can be distinguished from EC based on IP-10 and CCL4 (irrespective of stimulus), anti-PGL-I IgM and IL-10_WCS,_ therefore also showing significantly different median levels (Fig. 2, Table 1). Moreover, median levels of anti-PGL-I IgM and IP-10_Nil_ differed between MB patients and (BCG-vaccinated) HHC, whereas median levels of IP-10_WCS_ and CCL4_WCS_ only distinguished the non-vaccinated HHC from MB patients. BCG vaccination therefore affects the immune response in HHC, as reflected by the significant difference in IP-10_WCS_ levels between HHC and HHC&BCG (p = 0.018) (Fig. 2b). Furthermore, IL-10_WCS_ levels differed between HHC and EC, while median levels of IP-10_WCS_ and CCL4_WCS_ differed between HHC&BCG and EC as well. PB patients and EC showed significantly different median CCL4_Nil_ and CCL4_WCS_ levels (Fig. 2c), as well as borderline significant different levels for IL-10_WCS_ (p = 0.07) and IP-10_WCS_ (p = 0.06).

The ability of each analyte/stimulus combination to distinguish between two groups is summarized in Table 1, thereby reviewing the biomarker potential of the four individual host immune markers. Remarkably, the levels of IP-10 (p < 0, 0001), IL-10 (p = 0.003) and CCL4 (p < 0.0001) in WCS stimulated samples were more significantly different for MB and EC than anti-PGL-I IgM levels (p = 0.0042). Moreover, anti-PGL-I IgM levels could not be used to discriminate PB patients or (BCG-vaccinated) HHC from EC, which clearly demonstrates the added value of IP-10, IL-10 and CCL4 in leprosy diagnostics.

### Biomarker signatures to specify *M. leprae* infection, leprosy or disease classification

**Figure 3:**
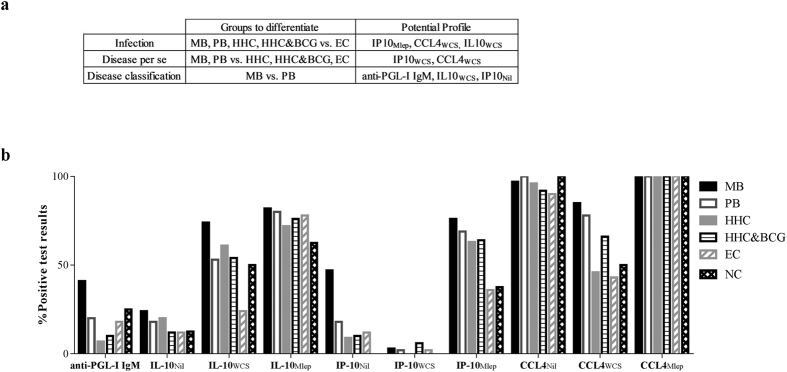
Positive test results per analyte/stimulus combination used to construct potential biomarker profiles. The groups that should be differentiated to indicate *M. leprae* infection, disease per se and disease classification are shown. The potential profiles indicated are based on the percentage of positive individuals of these particular groups. The cut-off for positivity was based on values for NEC (Supplementary Table S3) per analyte/stimulus combination the percentage of individuals with a positive test result per group is shown. Based on these data the optimal analyte/stimulus combination to differentiate either infected from non-infected groups, patients and non-patients groups or MB and PB patients were selected to construct the potential profiles described.

Diagnostic tests that allow detection of *M. leprae* infection, leprosy per se and leprosy classification would be of great benefit to the general healthcare in leprosy endemic areas. The four host immune markers allowed distinction between two groups (Table 1). However, to distinguish *M. leprae* infected from non-infected individuals or patients from healthy contacts, we compared host immune markers for multiple groups (Fig. 3).

**Figure 4:**
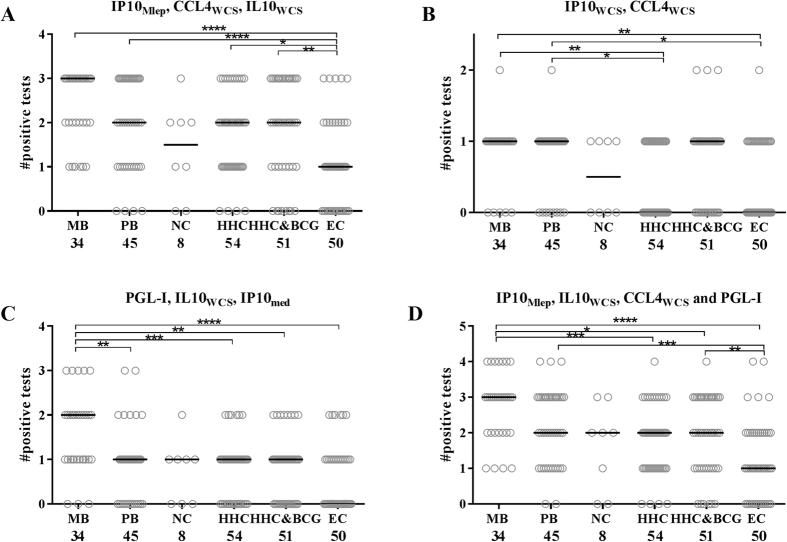
Potential of biomarker profiles to indicate *M. leprae* infection, disease per se and disease classification. The amount of positive test results per group is shown. (**a**) IP-10_Mlep_, CCL4_WCS_ and IL-10_WCS_ significantly differed in MB/PB patients and (BCG-vaccinated) HHC from EC, showing more positive test results in the groups that are exposed to *M. leprae* and thereby indicating *M. leprae* infection. (**b**) CCL4_WCS_ and IP-10_WCS_ enabled the distinction between patients and HHC, thereby indicating the pathogenic immune responses to *M. leprae* in patients. (**c**) Anti-PGL-I IgM, IL-10_WCS_ and IP-10_Nil_ showed more positive test results in MB patients thereby enabling the distinction between MB and PB patients. (**d**) A four marker profile of IL-10_WCS_, IP-10_Mlep_, CCL4_WCS_ and anti-PGL-I IgM shows the majority of significant differences observed in A, B and C. P-values: *p ≤ 0.05, **p ≤ 0.01, ***p ≤ 0.001, ****p ≤ 0.0001.

First, in order to combine immune markers into multicomponent host biomarker profiles, positive UCP-LFA results for each analyte/stimulus combination were collectively specified (Fig. 3; Supplementary Table S3). Second, analyte/stimulus combinations were selected such that they optimally distinguished individuals with a specified disease- or infection state (Fig. 3), considering all HHC as *M. leprae* infected. This resulted in three specific profiles:

- To indicate *M. leprae* infection we selected single test results obtained with IP-10_Mlep_, CCL4_WCS_ and IL-10_WCS_ UCP-LFAs as these analyte/stimulus combinations individually showed the least positive test results for EC compared to the *M. leprae* infected test groups (MB, PB, HHC and HHC&BCG) (Fig. 3). The combination of IP-10_Mlep_, CCL4_WCS_ and IL-10_WCS_ indeed was more frequently positive for MB/PB patients and (BCG-vaccinated) HHC than EC (Fig. 4a). Moreover, AUCs confirmed discrimination between non-infected and *M. leprae* infected test groups based on this multicomponent host immune profile (AUCs: 0.84 (MB vs. EC), 0.75 (PB vs. EC), 0.7 (HHC vs. EC) and 0.71 (HHC&BCG vs. EC) (Supplementary Table S4A).
- To detect leprosy patients from healthy, though possibly *M. leprae* infected individuals, CCL4_WCS_ and IP-10_WCS_ were selected as immune markers since these single tests were more frequently positive in patients (MB and PB) compared to contacts (HCC and HCC&BCG) and are therefore associated with pathogenic immunity to *M. leprae*. The combination of CCL4_WCS_ and IP-10_WCS_ indeed demonstrated a positive test result more often in patients than in HHC or EC (Fig. 4b), whereas the related AUCs were ≥0,66 thus confirming leprosy disease-specificity (Supplementary Table S4B).
- For classification of leprosy a signature consisting of anti-PGL-I IgM, IL-10_WCS_ and IP-10_Nil_ was applied, as each of these markers individually showed more positive test results in MB patients compared to PB patients (Fig. 3). This profile proved to be specific for MB patients (Fig. 4c) and thereby allowed the differentiation of MB and PB patients (AUC = 0.73, Supplementary Table S4C).

Ideally only one multicomponent host biomarker profile for diagnosis of *M. leprae* infection, leprosy per se and leprosy classification would be more suitable for field-use. In this exploratory study, a 4 marker profile of IL-10_WCS_, IP-10_Mlep_, CCL4_WCS_ and anti-PGL-I IgM was selected for this purpose, enabling distinction of infected and non-infected individuals by IL-10_WCS_, IP-10_Mlep_ and CCL4_WCS_, MB and PB patients from HHC and EC by CCL4_WCS_ and MB from PB patients by anti-PGL-I IgM and IL-10_WCS_ (Fig. 4d; Supplementary Table S4D). However, to distinguish MB from PB patients or PB patients from HHC profile III for leprosy classification showed a higher AUC compared to the 4 marker profile (0.73 vs. 0.65 and 0.66 vs. 0.62 respectively, Supplementary Table S4). These data indicate the importance of distinct phase-specific profiles, the application of which will depend on the nature of the diagnosis to be made.

Nonetheless, application of the 4 marker profile demonstrated the influence of multicomponent host biomarker profiles on test accuracy, showing increased AUCs compared to individual markers (Supplementary Figure S1). The added value of using various analytes indicates the potential of multicomponent host biomarker profiles for leprosy diagnostics to detect *M. leprae* infection, leprosy disease or disease classification.

## Discussion

The obvious incessant transmission of *M. leprae* has brought about increased focus in leprosy research on discovery of biomarkers to improve diagnosis. Nevertheless, thus far only few biomarkers for leprosy are recommended by expert panels^11^. Consequently, there is a growing need for new and sensitive diagnostic tools based on specific biomarkers which should, ideally, allow straightforward translation into field-friendly tests.

In this exploratory study, we aimed to provide several multicomponent host immune-biomarker profiles which distinguish between distinct stages of *M. leprae* infection. In this process we also emphasized the challenges that need to be tackled to allow application of these biomarkers in the field. As high-tech laboratories are often lacking in leprosy endemic areas, we examined the diagnostic potential of earlier developed field-friendly UCP-LFAs for detection of anti-PGL-I IgM antibodies and cyto/chemokines IP-10, IL-10 and CCL4^24^,^28^,^31^, in an extensive cohort in Bangladesh.

We demonstrated the biomarker potential of IP-10, IL-10, CCL4 and anti-PGL-I IgM measured by UCP-LFAs in whole blood, either in response to *M. leprae* specific stimuli or without stimulus. Moreover, multicomponent host biomarker profiles including selected analyte/stimulus combinations could indicate *M. leprae* infection, leprosy per se or be used for classification of leprosy subtypes. A biomarker profile of IP-10_Mlep_, CCL4_WCS_ and IL-10_WCS_ was highly indicative of *M. leprae* infection, consistent with our previous finding that the IP-10 response to *M. leprae* specific proteins indicates exposure to *M. leprae*^16^,^32^.

Leprosy per se, on the other hand, was indicated by CCL4_WCS_ and IP-10 _WCS_, showing the potential to identify pathogenic immunity against *M. leprae* and confirming earlier observations on CCL4^16^. As current diagnostic assays for leprosy are antibody-based and only facilitate the diagnosis of MB cases^33^,^34^,^35^, inclusion of the host immune markers CCL4 and IP-10 in the profile shows promise for diagnosis of PB patients and indicates the importance of measuring cellular markers simultaneously with humoral markers.

For leprosy classification, the combination of anti-PGL-I IgM, IL-10_WCS_ and IP-10_Nil_ was indicative for MB patients, enabling the distinction between MB and PB patients. Although IL-10 and particularly anti-PGL-I IgM have been identified as characteristic markers for MB leprosy^36^,^37^, we also identified IP-10 as a, seemingly counterintuitive, host immune marker for patients at this side of the spectrum who usually display decreased pro-inflammatory immunity. However, since T-cells are not the exclusive source of IP-10^38^, IP-10 may still be produced in MB patients by monocytes and neutrophils^39^, as described for HIV-infected TB patients^40^.

To detect *M. leprae* infection, leprosy per se, as well as leprosy classification simultaneously with only one biomarker profile, IL-10_WCS_, IP-10_Mlep_, CCL4_WCS_ and anti-PGL-I IgM demonstrated the most optimal 4 marker profile performance. However, it performed less optimal for the distinct stages of *M. leprae* infection than the phase-specific profiles. Other cyto-/chemokines to identify pathogenic immunity to *M. leprae* (e.g. MCP-1 and IL-1β^16^), leprosy classification (e.g. CCL17 and CCL18^41^) or general mycobacterial infection (EN-RAGE^42^,^43^) could therefore be included to achieve more optimal diagnostic accuracy^44^ as distinct phase-specific profiles. In a multiplex UCP-LFA format multicomponent host immune biomarker profiles can be measured in one single test. This format therefore provides a field-friendly diagnostic tool, facilitating the diagnosis of leprosy based on biomarker signatures.

Of note is the observation that CCL4 levels in response to *M. leprae* WCS were elevated for HHC who received BCG vaccination compared to those who did not. Thus, BCG vaccination may also cause increased pro-inflammatory immune responses which renders contacts more prone to development of over-reactive, pathogenic immunity to *M. leprae*. Indeed, in a recent vaccination study an unexpectedly high proportion of HHC presented with PB leprosy after BCG vaccination supporting this idea^45^. In this respect, this vaccination study also shows the importance of immunomonitoring individuals at high risk to identify and treat patients at an early stage. In addition, since BCG vaccination or boost is a well-accepted prophylaxis against leprosy in contacts of newly diagnosed patients^46^, it is relevant to distinguish BCG-induced immunity in healthy contacts from early stage leprosy in these individuals. To efficiently monitor contacts for this purpose, the different stages of infection and disease of leprosy should be covered in diagnostics tools. Through simultaneous measurement of all analytes of interest on a single lateral flow strip, this format allows assessment of multicomponent host biomarker profiles using a unique field-friendly technology^24^,^29^,^31^. Thereby, the UCP-LFA format not only provides diagnostic tools for leprosy but similarly holds promise for TB diagnosis^28^ and immunomonitoring of other chronic diseases^31^.

## Materials and Methods

### Study participants

Participants were recruited on a voluntary basis between January 2013 and December 2014 in leprosy endemic areas in Bangladesh as described previously^30^. Leprosy was diagnosed based on clinical, bacteriological and histological observations and classified by skin smears according to Ridley and Jopling^14^. Clinical and demographic data was collected in a database. Participants were classified into six test groups; MB patients, PB patients, HHC, HHC&BCG, NC and EC. Control individuals from the same leprosy endemic area (EC) were examined for the absence of clinical signs and symptoms of leprosy and TB; staff of leprosy- or TB clinics were excluded.

### Test group selection

A randomized sample selection was taken from 1110 participants^30^. Individuals were randomly assigned for sample inclusion using the RAND formula (Excel 2010), aiming for a 50/50 male/female ratio and a 1:1:1 ratio of three age groups: 0–14, 15–29, and 30+ (Supplementary Table S1). In total 242 individuals were selected; MB patients (n = 34), PB patients (n = 45), HHC (n = 54), HHC&BCG (n = 50), EC (n = 51) and NC (n = 8; PB = 7, MB = 1). Patient characteristics are shown in Supplementary Table S2.

### Leprosy prevalence

During this study the prevalence in the four districts (Nilphamari, Rongpur, Ponchagor en Thakurganch) was 0.82 per 10,000 with a new case detection rate of 0.98 per 10,000 (monthly report of Rural Health Program of 4 districts of Nilphamari, Bangladesh).

### Whole blood assay (WBA)

Upon recruitment venous, heparinized blood (4 ml) was used directly in whole blood assays (WBA), using microtubes pre-coated with *M. leprae* whole cell sonicate (designated WCS), ML2478/ML0840 recombinant proteins (designated Mlep)^16^ or without antigen stimulus (designated Nil)^30^. After 24 hour incubation at 37 °C materials were frozen at −20 °C, shipped on dry ice to the LUMC and stored at −80 °C until analysis by ELISA or UCP-LFA^24^.

### PGL-I and *M. leprae* whole cell sonicate (WCS)

Synthesized disaccharide epitope (3,6-di-O-methyl-β-D-glucopyranosyl(1 → 4)2,3-di-O-methylrhamnopyranoside), similar to *M. leprae* specific PGL-I glycolipid, coupled to human serum albumin (synthetic PGL-I; designated ND-O-HSA) and *M. leprae* whole cell sonicate (WCS) generated with support from the NIH/NIAID Leprosy Contract N01-AI-25469 were obtained through the Biodefense and Emerging Infections Research Resources Repository (http://www.beiresources.org/TBVTRMResearchMaterials/tabid/1431/Default.aspx)^47^.

### PGL-I ELISA

IgM antibodies against *M. leprae* PGL-I were detected as previously described^19^. Absorbance of horseradish peroxidase (HRP) was determined at a wavelength of 450 nm.

### ELISA for IL-10, IP-10 and CCL4

IP-10 (851.870.015, Diaclone Research, Besancon, France), IL-10 (851.540.015, Diaclone Research, Besancon, France) and CCL4 (DY271-05, R&D systems, Minneapolis, USA) ELISA kits were used. ELISA testing was performed according to the manufacturer’s protocol using coating antibody clones B-S10, B-C50 and #24006 and detection antibodies B-T10, BC-55 and BAF271 respectively for IL-10, IP-10 and CCL4. HRP absorbance was determined at wavelength of 450 nm.

### UCP-LFA for IL-10, IP-10 and CCL4

UCP-LFAs for CCL4, IL-10 and IP-10 were prepared and performed as described previously^24^,^28^,^29^. The same antibody pairs as used for ELISAs were applied, with the non-biotinylated variant of the detection antibodies (non-biotinylated CCL4: AF-271-NA). Briefly, mixtures of 100 ng cytokine-specific UCP reporter conjugate and diluted serum sample (1:4 for IL-10, 1:30 for IP-10 and 1:300 for CCL4) were incubated for 60 min on a thermoshaker at 37 °C and 900 rpm. The mixture was then applied to cytokine-specific LF strips (containing a Test line with an antibody complementary to the antibody on the UCP particles) and immunochromatography was allowed to continue until strips were dry. LF strips were scanned in a Packard FluoroCount microtiterplate reader adapted for measurement of the UCP label (980 nm IR excitation, 550 nm emission). Results are displayed as the ratio value between Test and Flow-Control signal based on relative fluorescence units (RFUs) measured at the respective lines^48^. Ratio values were translated to concentration based on standard curves for each immunemarker. Lower limit of detection was 32 pg/ml for IL-10 and 316 pg/ml for IP-10 and CCL4.

To determine test positivity, similar wholeblood samples from a set of healthy, non-endemic control individuals (NEC) were analysed and UCP-LFA thresholds were calculated based on the average value of all NEC samples (Supplementary Table S3).

### UCP-LFA for anti-PGL-I antibody

For detection of anti-PGL-I IgM antibodies, the same protocol as used for cytokine detection was applied utilizing 100-fold diluted serum and IgM-specific UCP conjugate (UCP^αIgM^). Only unstimulated samples were analysed as the level of antibody levels does not change upon antigen stimulation. The threshold for positivity of 0.29 was determined by computing receiver operating characteristic (ROC) curves.

### Ethics

This study was performed according to the Helsinki Declaration as described previously^30^. The national Research Ethics Committee (Bangladesh Medical Research Council) has approved the study protocol (Ref no. BMRC/NREC/2010-2013/1534).

Participants were informed about the study-objectives, the samples and their right to refuse to take part or withdraw from the study without consequences for their treatment. Written informed consent was obtained before enrolment. All patients received treatment according to national guidelines.

### ROC curves

Graphpad Prism version 6.02 for Windows (GraphPad Software, San Diego CA, USA) was used to plot ROC curves and calculate the area under curve (AUC); for IP-10, IL-10, and CCL4 the concentrations (pg/ml) were applied, whereas for anti-PGL-I IgM the OD_450_ corrected for background (ELISA) and ratio value (UCP-LFAs) was used.

### Statistical analysis

Differences in cytokine or antibody levels between test groups, as determined with UCP-LFA, were analysed with the One-way ANOVA for non-parametric distribution (Kruskall-Wallis) and Dunn’s correction for multiple testing using GraphPad Prism. For IP-10, IL-10 and CCL4 the concentrations (pg/ml) and for anti-PGL-I IgM the UCP-LFA ratio values were utilized. The statistical significance level used was p ≤ 0.05.

## Additional Information

**How to cite this article**: van Hooij, A. *et al*. Quantitative lateral flow strip assays as User-Friendly Tools To Detect Biomarker Profiles For Leprosy. *Sci. Rep.* **6**, 34260; doi: 10.1038/srep34260 (2016).

## Supplementary Material

Supplementary Information

## Figures and Tables

**Table 1:** Discriminatory biomarkers with potential for leprosy diagnostics.

	MB vs HHC	MB vs HHC&BCG	MB vs EC	PB vs EC	HHC vs EC	HHC&BCG vs EC	HHC&BCG vs HHC
anti-PGL-I IgM	++	++	++	−	−	−	−
IP-10	++	++	++	−	−	−	−
IP-10 WCS	++++	−	++++	−	−	++	+
IP-10 *M. leprae* proteins	−	−	+	−	−	−	−
IL-10	−	−	−	−	−	−	−
IL-10 WCS	−	−	+++	−	++	+	−
IL-10 *M. leprae* proteins	−	−	−	−	−	−	−
CCL4	−	−	+	+	−	−	−
CCL4 WCS	++	−	++++	+	−	++	−
CCL4 *M. leprae* proteins	−	−	+	−	−	−	−

Differences in IP-10, IL-10, CCL4 and anti-PGL-I IgM levels between various test groups detected by UCP-LFA are provided. Each row represents one of the 10 different analyte/stimulus combinations measured. Each column shows the potential to distinguish the test groups indicated, only displaying the groups for which significant differences were observed: −p ≥ 0.05 indicates inability to distinguish test groups, +p ≤ 0.05, ++p ≤ 0.01, +++p ≤ 0.001, ++++p ≤ 0.0001 indicating increasing capacity to distinguish test groups. Using one or multiple analyte/stimulus combination MB patients could be distinguished from (BCG-vaccinated) HHC and EC, whereas PB patients and BCG-vaccinated HHC could be distinguished from EC.
