# Supplementary material for: Quantitative lateral flow strip assays as User-Friendly Tools To Detect Biomarker Profiles For Leprosy
Source: Sci Rep. 2016 Sep 29;6:34260. doi: 10.1038/srep34260 (PMC5041085; doi:10.1038/srep34260)
Supplement: Supplementary Information [file srep34260-s1.pdf]

SUPPLEMENTARY INFORMATION

QUANTITATIVE LATERAL FLOW STRIP ASSAYS AS USER-FRIENDLY TOOLS TO DETECT BIOMARKER PROFILES FOR LEPROSY

Anouk van Hooij[*], Elisa M. Tjon Kon Fat[¶], Renate Richardus[♦], Susan J.F. van den Eeden[*], Louis Wilson[*], Claudia J. de Dood[¶], Roel Faber[♦], Korshed Alam[§], Jan Hendrik Richardus[♦], Paul L.A.M. Corstjens[¶] and Annemieke Geluk[*]

From the [*]Dept. of Infectious Diseases and [¶] Dept. Molecular Cell Biology, Leiden University Medical Center, The Netherlands, [♦]Department of Public Health, Erasmus MC, University Medical Center Rotterdam, Rotterdam, The Netherlands, [§]Rural Health Program, The Leprosy Mission International Bangladesh, Nilphamari, Bangladesh

Correspondence to: Prof. dr. A. Geluk, Dept. of Infectious Diseases, LUMC

PO Box 9600, 2300 RC Leiden, The Netherlands.

Tel: +31-71-526-1974 ; Fax +31-71-526-5267; E-mail: a.geluk@lumc.nl

Supplementary Table S1: *Test group selection*

Test group	Gender	Age group		
		**0-14**	**15-29**	**30+**
EC	F	10	10	10
EC	M	10	10	10
HHC&BCG	F	10	10	10
HHC&BCG	M	10	10	10
HHC	F	10	10	10
HHC	M	10	10	10
MB	F	2	1	4
MB	M	0	4	20
PB	F	2	8	11
PB	M	0	15	15

Samples were randomly selected using a 50/50 ratio of males (M) and females (F) and a 1:1:1 ratio of three age groups (0-14 yrs; 15-29 yrs; 30 yrs or older) within each group. For the MB and PB patient groups samples could not be evenly distributed for age and gender, due to the number of samples present.

Supplementary Table S2: *Patient characteristics*

	BI[1]	Classification[2]
MB1	5	BL
MB2	5	LL
MB3	4	BL
MB4	4	BL
MB5	4	LL
MB6	4	LL
MB7	3	BL
MB8	3	BT
MB9	1	BT
MB10	0	BL
MB11	0	BT
MB12	0	BT
MB13	0	BT
MB14	0	BT
MB15	0	BT
MB16	0	BT
MB17	0	BT
MB18	0	BT
MB19	0	BT
MB20	0	BT
MB21	0	BT
MB22	0	BT
MB23	0	BT
MB24	0	BT
MB25	0	BT
MB26	0	BT
MB27	0	BT
MB28	0	BT
MB29	0	BT
MB30	0	BT
MB31	0	BT
MB32	0	BT
MB33	0	BT
MB34	0	BT

	Classification
PB1	TT
PB2	TT
PB3	TT
PB4	TT
PB5	BT
PB6	BT
PB7	BT
PB8	BT
PB9	BT
PB10	BT
PB11	BT
PB12	BT
PB13	BT
PB14	BT
PB15	BT
PB16	BT
PB17	BT
PB18	BT
PB19	BT
PB20	BT
PB21	BT
PB22	BT
PB23	BT
PB24	BT
PB25	BT
PB26	BT
PB27	BT
PB28	BT
PB29	BT
PB30	BT
PB31	BT
PB32	BT
PB33	BT
PB34	BT
PB35	BT
PB36	BT
PB37	BT
PB38	BT
PB39	BT
PB40	BT
PB41	BT
PB42	BT
PB43	BT
PB44	BT
PB45	BT

[1] *The bacterial index (BI) is indicated for all MB patients.*

[2] *Patient characteristics according to Ridley-Jopling classification (1).*

Supplementary Table S3: *Cut-off values for IL-10, IP-10 and CCL4*

	IL-10 Nil	IL-10 WCS	IL-10 Mlep	IP-10 Nil	IP-10 WCS	IP-10 Mlep	CCL4 Nil	CCL4 WCS	CCL4 Mlep
NEC1	32	32	32	316	379	450	316	11621	1379
NEC2	32	32	32	316	1935	519	316	12732	566
NEC3	32	32	32	379	14771	790	316	8374	460
NEC4	32	32	32	1481	992	2015	316	2838	316
Threefold Average	96	96	96	1869	13557	2830	948	26673	680
Cut-off	100	100	100	1900	14000	2800	950	27000	700

IP-10, IL-10 and CCL4 concentrations (pg/ml) determined by UCP-LFA of 24h whole blood cultures of non-endemic healthy control individuals (NEC; n=4) without stimulus (Nil), in response to M. leprae whole cell sonicate (WCS) or M. leprae recombinant proteins ML2478 and ML0840 (Mlep). The cut-off for a positive test result was set as three times the average value of the four NEC in unstimulated samples, WCS and Mlep stimulated samples.

Supplementary Table S4: *Areas under the curve for each biomarker profile*

a

AUC	***M. leprae* Infection ($IP10_{Mlep}$ $CCL4_{WCS}$ $IL10_{WCS}$)**					
	MB	PB	NC	HHC	HHC&BCG	EC
MB		ns	0,76	0,7	0,63	0,84
PB	ns		ns	ns	ns	0,75
NC	0,76	ns		ns	ns	ns
HHC	0,7	ns	ns		ns	0,7
HHC&BCG	0,63	ns	ns	ns		0,71
EC	0,84	0,75	ns	0,7	0,71	

b

AUC	**Leprosy ($IP10_{WCS}$ $CCL4_{WCS}$)**					
	MB	PB	NC	HHC	HHC&BCG	EC
MB		ns	ns	0,7	ns	0,71
PB	ns		ns	0,66	ns	0,67
NC	ns	ns		ns	ns	ns
HHC	0,7	0,66	ns		0,61	ns
HHC&BCG	ns	ns	ns	0,61		0,62
EC	0,71	0,67	ns	ns	0,62	

c

AUC	**Leprosy Classification (PGL-I $IL10_{WCS}$ $IP10_{med}$)**					
	MB	PB	NC	HHC	HHC&BCG	EC
MB		0,73	0,77	0,77	0,73	0,82
PB	0,73		ns	ns	ns	0,64
NC	0,77	ns		ns	ns	ns
HHC	0,77	ns	ns		ns	0,61
HHC&BCG	0,73	ns	ns	ns		0,63
EC	0,82	0,64	ns	0,61	0,63	

d

AUC	**Four marker profile ($IP10_{Mlep}$ $IL10_{WCS}$ $CCL4_{WCS}$ PGL-I)**					
	MB	PB	NC	HHC	HHC&BCG	EC
MB		0,65	0,77	0,77	0,7	0,86
PB	0,65		ns	0,62	ns	0,75
NC	0,77	ns		ns	ns	ns
HHC	0,77	0,62	ns		ns	0,67
HHC&BCG	0,7	ns	ns	ns		0,69
EC	0,86	0,75	ns	0,67	0,69	

Areas under the curve (AUC) were determined using Graphpad Prism version 6.02 for Windows (GraphPad Software, San Diego CA, USA) for M. leprae infection (a), leprosy (b), leprosy classification (c) and the four marker profile (d), displaying only AUCs with a significant p-value

(ns=non-significant). AUCs were determined for each combination of groups. (MB: multibacillary patients; PB: paucibacillary patients; NC=new cases who developed leprosy after BCG vaccination; HHC: healthy household contacts; HHC&BCG: BCG-vaccinated HHC; EC: endemic controls). (a) Combined test results for IP-10$_{Mlep}$, CCL4$_{WCS}$ and IL10$_{WCS;}$ (b) Combined test results for IP-10$_{WCS}$, and CCL4$_{WCS\ ;}$ (c) Combined test results for anti-PGL-I IgM IP-10$_{Med}$, and IL10$_{WCS;}$ (d) Four marker profile of IL-10$_{WCS}$, IP-10$_{Mlep}$, CCL4$_{WCS}$ and anti-PGL-I IgM, showing the potential of this profile to indicate M. leprae infection, leprosy per se and leprosy classification.

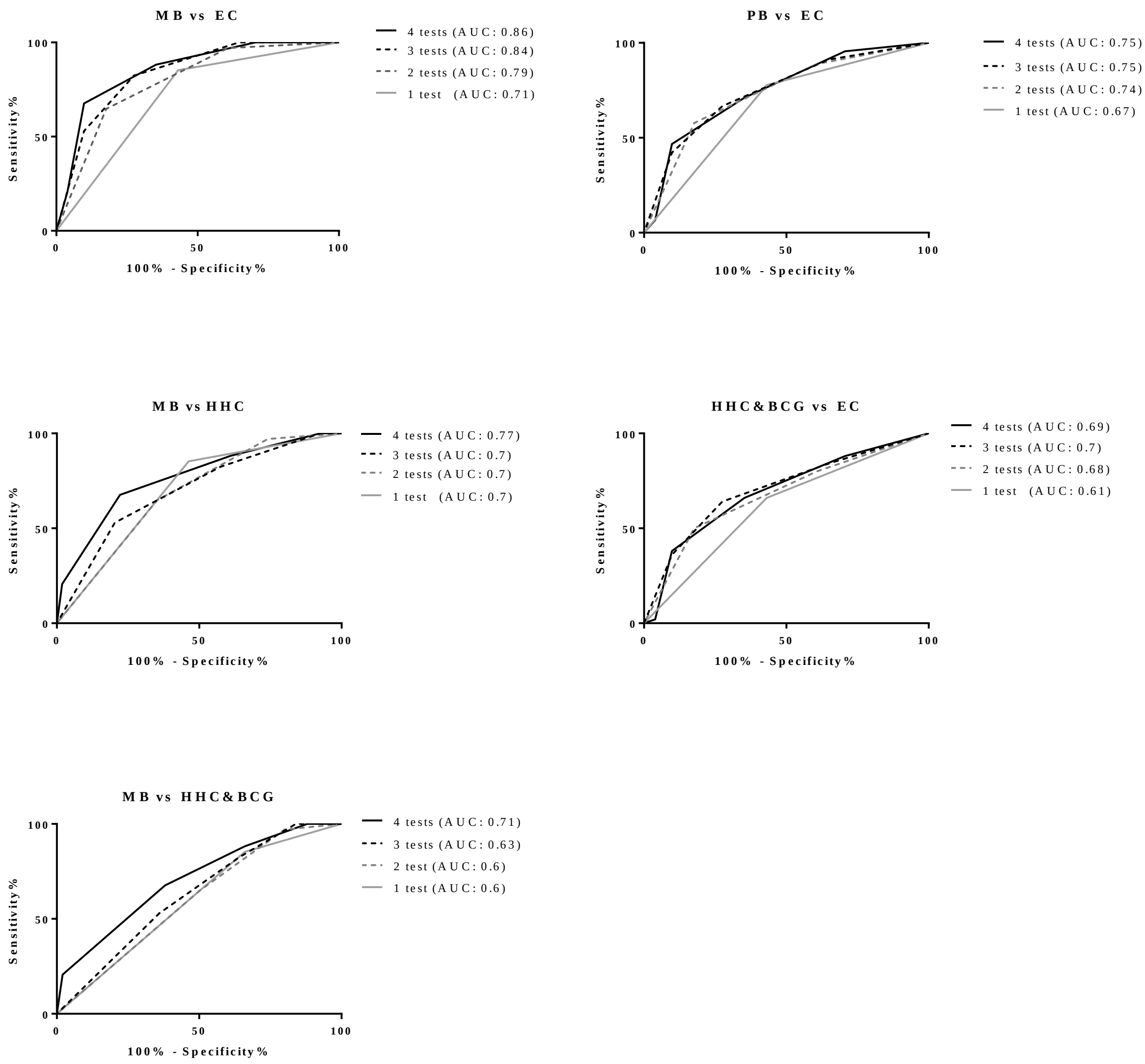

Supplementary Figure S1: ***Influence of multicomponent host biomarker profiles on test accuracy.***

ROC curves and the corresponding areas under the curve (AUC) using UCP-LFAs for 1, 2, 3 or 4 marker profiles (CCL4$_{WCS}$, IP-10$_{Mlep}$, IL-10$_{WCS}$ and anti-PGL-I IgM). Only the groups that showed significant differences in test results based on this 4 marker profile are shown. The AUC for the 4 marker profile test was increased compared to the AUC for 1 marker profile, showing improved diagnostic accuracy (2).

1 marker profile = $CCL4_{WCS}$ (the most sensitive test condition for detecting patients); 2 marker profile = $CCL4_{WCS}$ and $IP\text{-}10_{Mlep}$; 3 marker profile = $CCL4_{WCS,}$ $IP\text{-}10_{Mlep}$ and $IL\text{-}10_{WCS}$; 4 marker profile = $CCL4_{WCS,}$ $IP\text{-}10_{Mlep}$, $IL\text{-}10_{WCS}$ and anti-PGL-I IgM.

Reference List

1. Ridley, D. S., and W. H. Jopling. 1966. Classification of leprosy according to immunity. A five-group system. *Int. J. Lepr. Other Mycobact. Dis.* 34: 255-273.

2. Zou, K. H., A. J. O'Malley, and L. Mauri. 2007. Receiver-operating characteristic analysis for evaluating diagnostic tests and predictive models. *Circulation* 115: 654-657.
